# Editorial: Risk communication and community engagement during public health emergencies

**DOI:** 10.3389/fpubh.2023.1164973

**Published:** 2023-03-23

**Authors:** Guangyu Hu, Zhuo Chen, Jing Wang, Suli Huang

**Affiliations:** ^1^Institute of Medical Information/Center for Health Policy and Management, Chinese Academy of Medical Sciences and Peking Union Medical College, Beijing, China; ^2^Department of Health Policy and Management, College of Public Health, University of Georgia, Athens, GA, United States; ^3^School of Economics, Faculty of Humanities and Social Sciences, University of Nottingham Ningbo China, Ningbo, Zhejiang, China; ^4^School of Public Health and Management, Hubei University of Medicine, Shiyan, Hubei, China; ^5^Shenzhen Center for Disease Control and Prevention, Shenzhen, China

**Keywords:** risk communication, community engagement, public health emergencies, pandemic, COVID-19

## Introduction

During the early stage of the COVID-19 pandemic, the World Health Organization recommended including risk communication and community engagement (RCCE) as part of national public health emergency responses (1). As the pandemic evolves, effective communication with the public through reliable channels and engaging the public has gradually been recognized as equally important as developing appropriate containment measures and recommendations for policymakers. How to integrate RCCE into national preparedness and response practice and ensure that it plays an essential role in a country's health emergency deserves an in-depth discussion with a multidisciplinary perspective.

This Research Topic on “Risk communication and community engagement during public health emergencies” contains a collection of research articles that focus on lessons learned from RCCE response strategies in different nations. It provides insights into global comparisons of preparedness and response strategies across countries and pandemic management realities in different regions. The disproportionate impact of the pandemic and containment measures on vulnerable groups was empirically analyzed and shared. Under the compound risk scenario, a risk communication case study was also included. The current Research Topic consists of seven research articles that examined ways for nations to enhance RCCE during a pandemic, as well as perspectives of vulnerable groups during public health emergencies. We present the articles grouped into three themes in the following sections.

## Learning opportunities exist for countries to improve RCCE during a pandemic

The ability to develop evidence-based and effective RCCE responses to public health emergencies, including COVID-19, requires timely and comprehensive comparisons of preparedness and response strategies across nations. Brogan Geurts et al. from the Robert Koch Institute presented details of comparing governance structures for emergency risk communication in the early stages of the pandemic across Germany, Guinea, Nigeria, and Singapore. Their findings highlight the early integration of ECCE into preparedness and response plans, as well as lessons learned from previous experiences, which are necessary for effective resource use during the pandemic. In addition, this qualitative review highlights potential and transferable learning opportunities between countries with experience in outbreak management, particularly in bilateral communication and community engagement, as well as monitoring and evaluation.

Various health control mechanisms and public perceptions of inadequate health control in Latin American countries were examined in the study by researchers from the Universidad de Buenos Aires (Mejia et al.). This cross-sectional analytical study was conducted in 12 Latin American countries during the pandemic and shows that populations in multiple action scenarios experienced poor pandemic management. The results acknowledged that the most perceived inadequate control mechanisms established by a government were in Honduras, highlighting the unignorable health inequalities during the pandemic. Evidence on the associated factors would also help design effective mitigation plans for future pandemics and other emergencies.

Liu J. et al. from the Harbin Institute of Technology conducted a network analysis of China's central government's COVID-19 response by exploring the temporal characteristics of collaborative emergency information releases in public health emergencies. The Emergency Information Release Collaborative Networks (EIRCNs) and the Emergency Organizations-Emergency Information Release Matters (EOs-EIRMs) 2-mode network constructed in the research could help explain the driving factors and implementation mechanism of the temporal evolution characteristics of the collaborative emergency information release model and would help improve the efficiency of information release in other countries during a pandemic.

## Understanding vulnerable groups during public health emergencies

Some mitigation and containment measures have unintended consequences, particularly for historically marginalized racial and ethnic groups and health professionals. These communities would face disproportionate impacts of the pandemic (2), just as uptake of the COVID-19 vaccine has been unevenly impacted by vaccine hesitancy across different groups, and this is a pressing issue globally. Researchers from University College London explored the drivers of vaccine hesitancy in ethnic minority groups in the UK and focused on how vaccine hesitancy can be overcome (Naqvi et al.). The results highlight the important role that social media plays in vaccine hesitancy as well as identify enabling factors, such as a desire to travel and positive public health messages that can increase vaccine uptake among ethnic minority groups in the UK. Tailored public health messaging to address the concerns of marginalized communities by policymakers was also recognized as a critical issue.

Through a qualitative online survey, Ghirotto et al. from Azienda USL-IRCCS di Reggio Emilia investigated the opinions of Italian healthcare professionals who were part of categories affected by the country's mandatory COVID-19 vaccine regulations. The author considered the scientific evidence that drives ethics-related decisions and noted the epidemic of confusing and incorrect information affecting professionals. Increasing disaffection with the healthcare system and professional conflicts also highlights the fundamental role of RCCE in responding to emergencies, especially among healthcare professionals.

Wang and Wu from Fudan University shared an empirical case on providing basic necessities to vulnerable populations during the COVID-19 pandemic in Shanghai (Wang et al.). They reported key material supply problems, including a lack of procurement channels, insufficient material reserves, and insufficient transportation capacity, and provided tips and lessons learned on how to better manage the preparation, dispatch, and transportation of basic necessities that were in shortage for vulnerable populations during the city-wide lockdown in Shanghai.

## Taking the compound risk scenario into account

Zhang and Liu from Beijing Normal University put forward a new perspective on RCCE under the compound risk scenario (Liu C. et al.). They presented an empirical study of the effect of a flood event caused by extreme rainfall on COVID-19 prevention behaviors and found that the individual's flood risk perception and response behaviors were significantly correlated with the individual's COVID-19 prevention behaviors. They proposed that community risk preparedness behavior and social capital can moderate the above relationship to a certain extent. These findings would guide risk communication under the complex risk scenario and reduce risky public behaviors during a pandemic.

## Summary

As we read the articles included in this Research Topic, governments in different countries have adopted a range of strategies for RCCE, with profound consequences, both positive and negative. This Research Topic demonstrates that there are learning opportunities for countries to improve RCCE during a pandemic, and how vulnerable groups were affected and their perceptions varied. It also brought to our attention the complex risk scenario during emergencies and how to balance containment efforts with potentially unintended consequences. The contributions by authors and reviewers to this critical and timely topic are greatly appreciated. We hope this Research Topic will be useful to researchers and policymakers as it shares evidence-based insights on the COVID-19 pandemic from multinational academic research.

## Author contributions

GH, ZC, JW, and SH contributed to the conceptualization and design of the Research Topic and the editorial. GH wrote the first draft of the editorial. All authors contributed to the manuscript revision and approved the submitted version.
